# Supplementary material for: Concurrent Validity and Reliability of an Inertial Measurement Unit for the Assessment of Craniocervical Range of Motion in Subjects with Cerebral Palsy
Source: Diagnostics (Basel). 2020 Feb 1;10(2):80. doi: 10.3390/diagnostics10020080 (PMC7168926; doi:10.3390/diagnostics10020080)
Supplement: Supplementary file 1 [file diagnostics-10-00080-s001.pdf]

Table S1. Two-way ANOVA results for the spatial planes ROM by evaluation and group (CP: n=23; CG: n=23) factors*.

Spatial plane	Factor	Mean (95%CI)	p-value	Pair-wise comparison	Difference between means (95%CI)	p-value
**Flexion-extension**	**Evaluation**					
	CROM	131.7 (124.8; 138.6)	<0.001	*CROM vs IMU 1st assessment 1st day*	-4.1 (-6.2; -2.1)	<0.001
	IMU 1st assessment 1st day	135.8 (128.5; 143.1)		*CROM vs IMU 2nd assessment 1st day*	-5.8 (-11.8; -0.2)	0.049
	IMU 2nd assessment 1st day	137.4 (129.8; 145.2)		*CROM vs IMU 2nd day*	-9.5 (-17.7; -1.3)	0.015
	IMU 2nd day	141.2 (132.8; 149.6)		*IMU 1st assessment 1st day vs IMU 2nd assessment 1st day*	-1.6 (-8.0; 4.7)	0.989
				IMU 1st assessment 1st day vs IMU 2nd day	-5.4 (-13.2; 2.3)	0.160
				IMU 2nd assessment 1st day vs IMU 2nd day	-3.8 (-10.9; 3.4)	0.920
	**Group**					
	CP	135.5 (125.6; 145.3)	0.757		2.2 (-11.9; 16.2)	
	CG	137.6 (127.6; 147.7)				
**Rotational**	**Evaluation**					
	CROM	145.7 (141.1; 150.3)	0.020	*CROM vs IMU 1st assessment 1st day*	-4.5 (-10.4; 1.3)	0.218
	IMU 1st assessment 1st day	150.3 (144.5; 156.0)		*CROM vs IMU 2nd assessment 1st day*	-3.7 (-10.3; 2.9)	0.757
	IMU 2nd assessment 1st day	149.4 (143.4; 155.4)		*CROM vs IMU 2nd day*	-8.9 (-17.5; -0.4)	0.036
	IMU 2nd day	154.7 (148.9; 160.4)		*IMU 1st assessment 1st day vs IMU 2nd assessment 1º day*	0.8 (-5.0; 6.7)	0.990
				IMU 1st assessment 1st day vs IMU 2nd day	-4.4 (-13.7; 4.9)	0.980
				IMU 2nd assessment 1st day vs IMU 2nd day	-5.2 (-13.1; 2.6)	0.436
	**Group**					
	CP	151.5 (145.3; 157.9)	0.482		-3.1 (5.8; -12.0)	
	CG	148.5 (142.2; 154.7)				
**Side-bending**	**Evaluation**					
	CROM	95.7 (89.8; 102.0)	0.001	*CROM vs IMU 1st assessment 1st day*	4.5 (1.1; 8.0)	0.004
	IMU 1st assessment 1st day	91.1 (84.4; 97.5)		*CROM vs IMU 2nd assessment 1st day*	5.7 (1.2; 12.2)	0.019
	IMU 2nd assessment 1st day	89.9 (82.8; 94.8)		*CROM vs IMU 2nd day*	2.4 (0.0; 4.9)	0.048
	IMU 2nd day	93.5 (87.3; 97.5)		*IMU 1st assessment 1st day vs IMU 2nd assessment 1st day*	1.2 (-2.4; 6.8)	0.987
				IMU 1st assessment 1st day vs IMU 2nd day	-2.5 (-8.9; 3.4)	0.377
				IMU 2nd assessment 1st day vs IMU 2nd day	-3.4 (-8.7; 0.1)	0.061
	**Group**					
	CP	93,3 (85,1; 101,4)	0.725		-2.0 (-13.6; 9.5)	
	CG	91.3 (83.1; 99.4)				

* ANOVA evaluation-by-group interaction was not statistically significant (p>0.05).

Abbreviations: CROM, Cervical range of motion device; IMU 1st assessment 1st day, Inertial Measurement Unit first assessment on the first day; IMU 2nd assessment 1st day, Inertial Measurement Unit second assessment on the first day; IMU 2nd day, Inertial Measurement Unit assessment on the second day; CG, Control group; CP, Cerebral palsy group.

Values in parenthesis indicate the 95% confidence interval.

Data are expressed in degrees.
